# Improvement of serum folate status in the US women of reproductive age with fortified iodised salt with folic acid (FISFA study)

**DOI:** 10.1017/S1368980024001903

**Published:** 2024-10-24

**Authors:** Anastasia Arynchyna-Smith, Alexander N Arynchyn, Vijaya Kancherla, Kenneth Anselmi, Inmaculada Aban, Ron C Hoogeveen, Lyn M Steffen, David J Becker, Andrzej Kulczycki, Waldemar A Carlo, Jeffrey P Blount

**Affiliations:** 1 Division of Pediatric Neurosurgery, Department of Neurosurgery, University of Alabama at Birmingham, 1600 7th Ave South, JFL 400, Birmingham, AL 35233, USA; 2 Division of Preventive Medicine, Department of Medicine, University of Alabama at Birmingham, Birmingham, AL, USA; 3 Department of Epidemiology, Rollins School of Public Health, Emory University, Atlanta, GA, USA; 4 Department of Marketing and Supply Chain Management, College of Business, East Carolina University, Greenville, NC, USA; 5 Department of Biostatistics, School of Public Health, University of Alabama at Birmingham, Birmingham, AL, USA; 6 Department of Medicine, Baylor College of Medicine, Houston, TX, USA; 7 Division of Epidemiology and Community Health, University of Minnesota, Minneapolis, MN, USA; 8 Department of Health Policy and Organization, School of Public Health, University of Alabama at Birmingham, Birmingham, AL, USA; 9 Division of Neonatology, Department of Pediatrics, University of Alabama at Birmingham, Birmingham, AL, USA

**Keywords:** Neural tube defects, Folic acid, Serum folate concentration, Iodised salt, Prevention, Food fortification, Intervention, Spina bifida

## Abstract

**Objective:**

Mandatory folic acid fortification of enriched grains has reduced neural tube defect prevalence in several countries. We examined salt as an additional vehicle for folic acid fortification. The primary objective was to examine the change in serum folate concentration after 1 month of consumption of fortified iodised salt with folic acid (FISFA) among women of reproductive age. The secondary objectives were to examine (1) the feasibility of implementing FISFA intervention and (2) the acceptability of FISFA.

**Design:**

We conducted a pre–post intervention study (January–April 2023). Participants received a FISFA saltshaker with the study salt (1 g of sodium chloride salt fortified with 100 mcg of folic acid) to use instead of regular table salt for 1 month. Serum folate was measured using the Elecsys Folate-III immunoassay method at baseline and 1-month endpoint. Change in serum folate was assessed using a two-tailed Wilcoxon signed rank test for paired samples.

**Setting:**

Metropolitan city, Southern USA.

**Participants:**

Non-pregnant, 18–40-year-old women who lived alone/with a partner.

**Results:**

Thirty-two eligible women consented to participate, including eleven non-Hispanic-White, eleven non-Hispanic-Black and ten Hispanic. Post-intervention, there was a significant increase in median serum folate concentration of 1·40 nmol/l (IQR 0·74–2·05; *P* < 0·001) from 24·08 nmol/l to 25·96 nmol/l in an analytical sample of *n* 29. An increase was seen in 28/29 (93 %) participants. Feasibility: 100 % study consent and compliance. FISFA acceptability: 25 d average use; 1·28 g average daily intake; 96·7 % and 90 % reported taste and colour of FISFA as highly acceptable, respectively.

**Conclusions:**

FISFA is an effective approach to increasing serum folate concentrations among women of reproductive age. Findings should be replicated in a larger study.

## Graphical abstract

**Figure:**
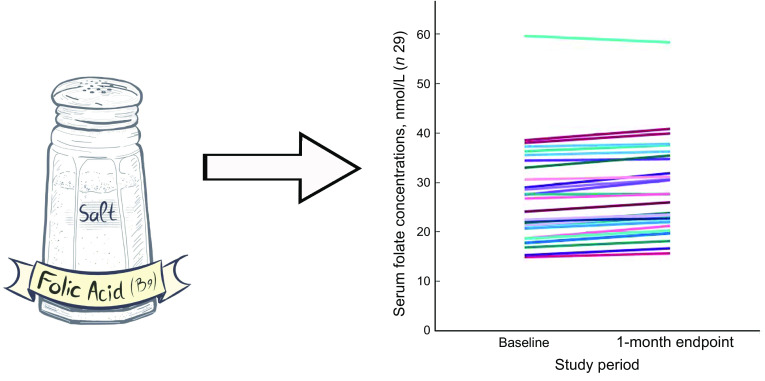

Neural tube defects (NTD) are the most common birth defects affecting the central nervous system. They occur during the earliest stages (around the 28th day after conception) of pregnancy before a woman is aware of conception, and they occur due to improper closure of the neural tube from which the brain and spinal cord later develop^(1,2)^. Every year, there are 214 000–322 000 new NTD worldwide, contributing to high mortality^(3)^. Children born with myelomeningocele, the most common type of NTD, often require extensive medical care with multiple surgeries, and many face life-long disability^(4,5)^. Quality-of-life concerns often include cognitive impairment, social stigmatisation/isolation, depression and inability to live independently^(6)^. Besides the NTD devastating effects, maternal health is also compromised through the increased risk of miscarriage, elective abortion, complex delivery and the physical and emotional toll of life-long care for a disabled child^(3,6–10)^. The most important epigenetic factor in NTD is maternal folate insufficiency^(2,11–13)^.

Folate is a water-soluble vitamin B_9_ that is found in dark green vegetables, fruits, nuts and legumes^(14)^. Folic acid (vitamin B_9_) is a synthetic form of folate. A dose–response relationship has been demonstrated between maternal erythrocyte folate concentration and NTD risk in the offspring^(15)^. The WHO guidelines recommend a minimum of 400 ng/ml (906 nmol/l) erythrocyte folate to optimise protection from NTD^(15)^. Notably, serum folate is involved in the direct transfer of folate to the fetus rather than from erythrocyte folate^(16)^. The desired protective sufficiency level in serum folate is 25·5 nmol/l (24·6, 26·4 95 % CI)^(17)^. Both serum and erythrocyte standards were derived through the microbiological assay method of analysis of folate^(15,17)^. There is no known cut-off value for high folate concentrations, and the literature has used 90th percentile as the arbitrary marker for upper-end values^(18)^.

Supplementation pills as a preventive strategy is not the most effective strategy because NTD occur very early in fetal development, and women are often unaware they are pregnant; therefore, they do not begin taking prenatal vitamins until a positive pregnancy test. Additionally, nearly half of all pregnancies in the USA are unplanned^(19)^. Further, a meta-analysis of folic acid supplementation during the preconception period showed that it is highly variable globally, and most women do not reach protective folate levels before conception^(20)^. Therefore, food fortification is the most effective strategy to protect against NTD, as it provides a passive, rather than reactive, method of ensuring adequate folate intake.

Salt offers a potential solution as a complementary and alternative food vehicle for folic acid fortification (FAF). Salt is a universal component of the diet globally and has long served as a vehicle in food fortification efforts. Iodisation programmes successfully laid the global infrastructure for salt fortification. Per Global Fortification Data Exchange, 126 countries have mandatory salt iodisation programmes, and an additional twenty-one countries have voluntary salt iodisation programmes^(21)^. This makes iodised salt a good vehicle for FAF in countries that need alternative vehicles to grains. In the USA, daily intake of salt ranges from 3·3 g to 9·2 g per capita per d and up to 70 % of salt is consumed from prepackaged foods^(22–25)^. Currently, salt in the USA is fortified with iodine at 76·5 mg/kg^(22)^. A recent study in India showed a significant increase in median folate levels among women of reproductive age (WRA) after 4 months of consumption of iodised salt fortified with folic acid^(26)^. Another ongoing study in India (NCT03853304) is assessing levels following folic acid and three other micronutrients^(27)^. Additionally, a three-arm randomised clinical trial is being conducted in Ethiopia to assess folate status after iodine only, iodine with low-dose folic acid and iodine with high-dose folic acid (NCT06223854), but, to our best knowledge, there are no other ongoing FAF trials.

Success in raising maternal folate concentrations has been seen in the USA, and some countries fortify enriched grain products (i.e. wheat flour) with folic acid^(28–31)^. In 1998, the USA implemented a successful mandatory policy on fortifying enriched grain products with 140 mcg of folic acid per 100 g^(32)^. A cross-sectional assessment of National Health and Nutrition Examination Survey (NHANES) data showed average serum folate concentrations significantly increased from 12 nmol/l in 1988 (pre-fortification) to 36 nmol/l in 2011–2016 (post-fortification) in the USA^(18,33)^. The prevalence of NTD declined from 10 (pre-fortification) to 7 (post-fortification) per 10 000 live births^(30)^. However, erythrocyte folate insufficiency in the USA persists in 20 % of WRA in the post-fortification era^(18)^. NHANES modelling study showed that 48 % of women in the USA consume folic acid only from enriched cereal grain products, and they have higher NTD prevalence compared with those who consume folic acid from additional sources^(34)^. The authors inferred that enriched grain may not be enough and recommended that it would be beneficial for this group to have another source of folic acid daily; therefore, salt with folic acid could be such a vehicle. Additionally, the US Preventive Services Task Force reaffirmed their recommendation of the need for an additional 400–800 mcg of folic acid supplement to combat the insufficiency rate^(35)^.

We identified the need to further improve folate concentrations in WRA in the USA. A recent study from the Centers for Disease Control and Prevention has shown that dietary folate is not sufficient to meet estimated average requirements and that folate levels can be increased through additional fortification; therefore, we wanted to look for a novel food vehicle^(19)^. The US setting ensured this study was conducted in a country with an active FAF policy of wheat flour, maize flour and rice^(22)^. The primary objective of this pre–post intervention study was to examine the change in serum folate concentration after 1-month of consumption of fortified iodised salt with folic acid (FISFA) among non-pregnant, non-lactating WRA. Secondary objectives included examining (1) the feasibility of implementing FISFA intervention and (2) the acceptability of FISFA.

## Methods

### Study design, setting and participants

The study used a pre–post intervention study design to examine the effectiveness of FISFA. The study was set at a large southeastern US university campus. Recruitment was conducted using flyers on campus, an email invitation sent to students and faculty at the School of Public Health and Health Professions and additional word-of-mouth communication by already enrolled participants. Potential participants reached out either via the REDCap interest survey or via email. Research staff conducted phone screening for eligibility and ability to comply with study procedures. Written informed consent was obtained from all participants. Weekly phone check-ins were made to verify usage of the study saltshaker and daily log. A final reminder about the endpoint visit was sent out. Participants included non-pregnant, non-lactating WRA (18–40 years) who did not intend to get pregnant and lived alone or with a partner without children for the duration of the study. We intended to enrol thirty-two women (eleven White, eleven Black, ten Hispanic) based on available study funding.

### Study salt (fortified iodised salt with folic acid)

The intervention consisted of the substitution of regular salt with a FISFA saltshaker for 1 month to use when cooking/eating at home or dorm and as a table salt when eating outside the home. FISFA samples were specifically produced for this study, and provided at no cost, by SWS AG (Südwestdeutsche Salzwerke AG, Bad Reichenhall), which had no role in the study design, data analysis or interpretation. Each gram of FISFA salt was fortified with 100 mcg of folic acid and 19 mcg of iodine. The colour of the salt had the expected light yellow tint attributable to the presence of folic acid. A daily serving of FISFA salt was set at 2 g based on a previous assessment of average salt intake of 9·2 g, of which 70 % comes from prepackaged foods^(22–24)^. This daily intake of 2 g of FISFA salt would correspond to the consumption of 200 mcg of folic acid. Guidance was provided on portion suggestions: 1/2 teaspoon, 2–3 pinches or forty shakes to help understand what one serving entails. Each FISFA container held approximately 125 g of salt. Additional or excessive salt intake was not promoted to participants. Participants filled out a daily paper log if they used FISFA for 1 month.

### Data collection

Demographic data, including sex, race, ethnicity, age, height, weight, insurance, home address, marital status, education level, employment status, income, rural–urban commuting area codes and Area Deprivation Index were collected via the participant-reported REDCap survey. History of previous pregnancies impacted by NTD in the participants, or a family history of NTD, Crohn’s disease, sickle cell anaemia and alcohol intake were also collected at baseline. The Willett FFQ was administered at baseline and 1-month endpoint to assess the amount of folate intake from the diet, intake of prenatal vitamins, multivitamins, folic acid supplementation pills and leading any special diet (vegetarian, vegan, gluten-free, etc.) for the past 30 d^(36)^. Paper FFQ surveys were de-identified with only the participant study identification number (ID) and mailed for analysis by the Harvard T.H. Chan School of Public Health Nutrition Questionnaire Service Center. Extrapolated data were emailed to the principal investigator via secure file transfer. All data were stored on the university’s network-protected REDCap server. All personal and health information was de-identified by a study ID.

### Assessment of participant characteristics and fortified iodised salt with folic acid intake at baseline and endpoint

Participants came to the study centre on campus at two time points, including the baseline and after 1 month (study endpoint). Both at the baseline and endpoint visits, participants filled out REDCap demographic and medical history surveys, on paper FFQ survey, and provided non-fasting blood samples. At baseline, they received a daily log to record salt use and received a saltshaker with FISFA. Then, at the 1-month endpoint, participants returned the completed daily log to record salt use and returned the FISFA saltshaker with any leftover salt. Participants received $100 for their time and effort.

Additionally, at baseline, each participant was handed a study folder that included (1) a copy of the consent form, (2) an infographic sheet on spina bifida, fortification with folic acid and global prevention statistics, (3) one page of daily salt log, (4) study magnet with saltshaker to hang the salt log on the fridge, (5) laminated door sign ‘Did I take my saltshaker?’ to hang on their front door to remember to take study salt with them when they left the house, (6) reminder sheet with instructions on what not and what to do regarding saltshaker care, (7) pen, (8) study contact information card, (9) payment card and (10) a piece of chocolate.

To assess the amount of FISFA intake during the study, each sealed FISFA saltshaker, including the salt, was weighed before study initiation by three study team members independently on the same scale and using the same calibrating process at the UAB lab. Participants returned the saltshakers to the study team after the completion of the study, at which point, they were weighed again by the same two study team members as at baseline, using the same scale and calibration process as used at the beginning of the study. The difference in weights of the saltshakers at baseline and endpoint was used to quantify the amount of salt consumed by each participant during the study period.

### Outcomes

The primary outcome of the study was the absolute change in measured serum folate concentrations in the 1-month interval from the baseline assessment to the post-intervention endpoint. Each participant acted as their control. Serum was chosen because it reflects the effect of recent folic acid intake in the body, whereas erythrocyte folate levels indicate the long-term intake, typically up to 6 months. Serum folate measures are more relevant at the time of neural tube development in the fetus, as serum folate freely binds to proteins (i.e. folate-binding protein and albumin) that are transferred from the mother to the fetus.

Secondary outcomes of interest in our study included study feasibility and acceptability of FISFA by study participants. Study feasibility was measured by (1) consent rate and (2) completion rates of study visits and dietary and lifestyle surveys. Acceptability of FISFA was measured by (1) completion of daily salt logs; (2) consumption of salt (by weighing saltshakers) and (3) attitudes towards the taste of the food when using FISFA and the colour of the FISFA. For assessing study feasibility, the consent rate was calculated by the total number of women who consented to the study divided by eligible women, and the completion rates of study visits and surveys were calculated by dividing baseline completion numbers by 1-month follow-up completion numbers. For assessing acceptability, the completion of daily salt logs was measured by counting the number of days salt was marked as consumed by the participant on the log. Consumption of salt was measured by weighing the saltshakers using the same properly recalibrated Fisher Science Education scale, model SLF302-US with an accuracy of 0·01 g, at baseline and at 1-month endpoint, and calculating the difference for each participant. Attitude towards taste was measured on a Likert scale by asking, ‘Did using this salt negatively affect the taste of your food overall?’ Attitude towards colour was measured on a Likert scale by asking, ‘Did the colour of salt negatively affect your desire to use it daily?’ Likert scale responses ranged from 1 to 5 from ‘Strongly disagree’ to ‘Strongly agree,’ with 1 being the most desirable answer.

### Laboratory procedures

Non-fasting blood samples were collected to test serum folate, erythrocyte folate and vitamin B_12_ concentrations at baseline and the 1-month post-intervention endpoint. Samples were drawn, processed and prepared for shipment by the UAB laboratory. Five tubes of blood samples (two serum separator [SST] tubes, two erythrocyte EDTA tubes and one haematocrit EDTA tube) were collected using standard phlebotomy procedures. SST tubes were collected first, followed by the EDTA tubes and finally by the haematocrit EDTA tube. Two millitres of blood was collected per tube for a maximum of 10 ml total. Each tube underwent mixing through seven inversions, with EDTA tubes placed on ice. For the SST tubes, a minimum of 30-min clotting period was observed, followed by centrifugation at 1300 ***g*** for 10 min at room temperature. After centrifugation, the serum was aliquoted in two cryogenic tubes (0·5 ml each) and frozen at –80°C. The erythrocyte EDTA tubes were immediately aliquoted in two cryogenic tubes (0·5 ml each) and frozen at –80°C. Haematocrit measurements (i.e. the ratio of erythrocyte to total blood volume expressed as a percentage (%) were conducted using the Hemoglobin Testing System (AimStrip Hb). After ensuring a proper device calibration, a blood drop was applied, providing Hb and estimated Hct values within 15 s. Results were recorded on Form DOPM-LAB-Form-006 Folic Acid Salt Lab form. All samples were de-identified prior to the shipment to a laboratory at Baylor College of Medicine and only indicated a participant ID to link each participant’s pre- and post-intervention samples.

Samples were analysed by the laboratory at Baylor College of Medicine. Serum folate concentrations were measured by the Roche Elecsys Folate-III immunoassay on a Cobas e411 immunoassay analyzer. This test has a testing range of 0·6–20 ng/ml where samples above 20 ng/ml were manually diluted with Elecsys Diluent Universal with a recommended dilution of 1:2 and up to 1:10. The maximum folate concentration that the test could capture was up to 453·0 nmol/l per the conversion rate of folate from ng/ml to nmol/l multiplied by 2·265 per WHO 2015 guidance^(37)^. Erythrocyte folate concentrations were measured by the Roche Elecsys Folate erythrocyte immunoassay on a Cobas e411 immunoassay analyzer according to manufacturer protocol. The assay has a measuring range of 120–620 ng/ml or 272–1407 nmol/l, where values above the measuring range can be manually diluted with a recommended dilution of 1:2. The maximum erythrocyte folate concentration that the assay could capture was up to 2814 nmol/l applying the conversion factor ng/ml × 2·265 = nmol/l. Serum vitamin B_12_ concentrations were measured by the Roche vitamin B_12_ electrochemiluminescence immunoassay on a Cobas e411 immunoassay analyzer. The Roche vitamin B_12_ assay has a measuring range of 22–1476 pmol/l or 30–2000 pg/ml, where values above the measuring range can be manually diluted with a recommended dilution of 1:2. The maximum vitamin B_12_ concentration that the test could capture was up to 2952 pmol/l applying the conversion factor pg/ml × 0·738 = pmol/l.

### Statistical analysis

Statistical analysis was conducted using SPSS 29·0 (SPSS Inc.). Frequencies and percentages were estimated in the initial descriptive analysis of participant characteristics. The normality of biomarker and dietary folate data was assessed using the Shapiro–Wilk test. Using the approach taken by Pfeiffer *et al*. (2019), we applied the 90th percentile cut-off value to our dataset to address extremely high folate values^(18)^. Mean and median serum folate concentrations at pre- and post-intervention up to the 90th percentile were compared using a two-tailed paired sample *t* test and Wilcoxon signed rank test for paired samples, respectively, with a significance value set at 0·05. The Clopper–Pearson method was used to calculate exact confidence intervals of the proportion of women who had increased their folate levels. Spearman correlation was computed to describe the association between the amount of FISFA intake and the magnitude of difference in serum concentrations pre- and post-intervention. Mean dietary folate intake at pre- and post-intervention were compared using a paired sample *t* test with significance set at 0·05. This study was conducted as proof-of-concept research. There was no previously published data on change in serum folate concentrations among WRA after intake of FISFA under an existing mandatory FAF context (e.g. the USA already has a FAF mandate) to our knowledge. The study required ten subjects to achieve 80 % power for detecting a significant effect with an alpha set at 0·05; however, we included three times more subjects in our analysis.

## Results

**Fig. 1:**
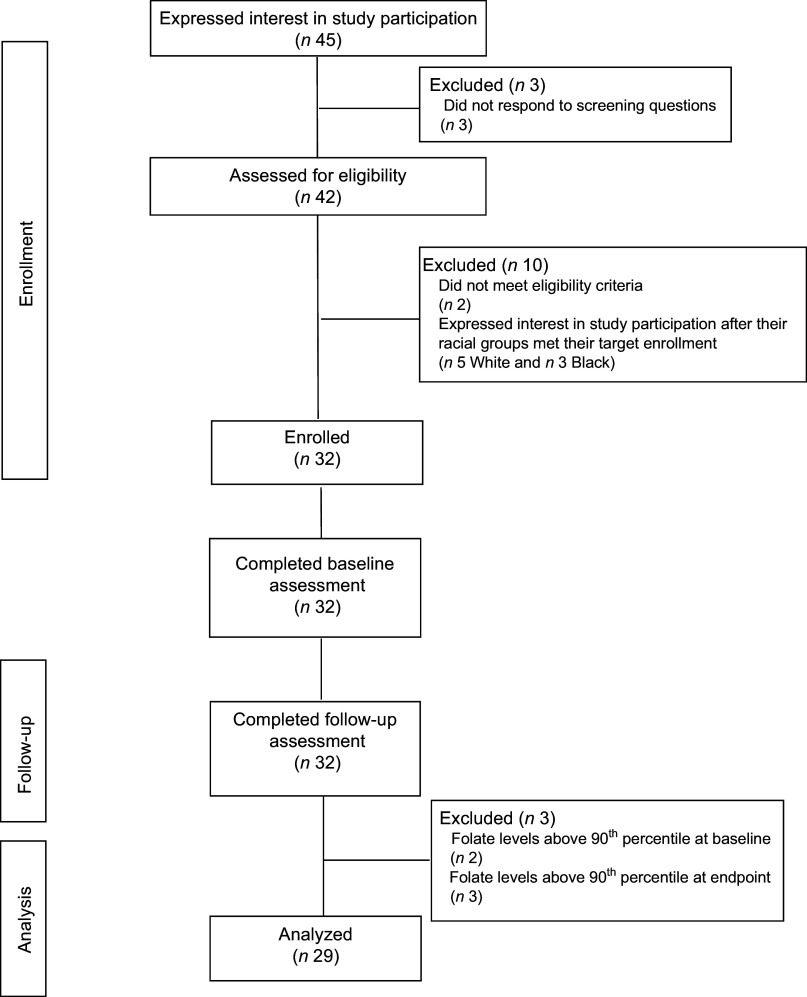
Consort diagram

A total of forty-five women were screened, forty were determined eligible and only thirty-two were recruited to participate in the study until race/ethnic groups were saturated due to limited study funding. Eight of the forty eligible women were placed ‘on reserve’ as we could only enrol thirty-two women adhering to the number of participants that we proposed in the grant protocol due to budget limitations (Fig. 1). Eight women who were not enrolled (five White non-Hispanic and three Black non-Hispanic) responded to the study flyer after race-specific groups were filled with 11 and 11, respectively, and the study continued to enrol Hispanic participants. The final analytic group consisted of eleven non-Hispanic White, eleven non-Hispanic Black and ten Hispanic White women. The mean age of the participants was 26·8 (sd 4·3) years; 94 % had completed higher education or a professional degree; 63 % were currently employed; and none had a history of NTD diagnosis nor had a pregnancy or family member affected by NTD (Table 1). Also, 66 % followed a non-special diet, 41 % took a daily multivitamin with folic acid and none took folic acid supplements alone (Table 2). Total dietary folate did not have normal distribution per the Shapiro–Wilk test; therefore, the median intake of total folate (dietary and supplemental) was 574 (IQR 372–887) mcg at baseline and 438 (IQR 299–866) mcg at the endpoint with a statistically significant decrease overall (–94 mcg; IQR –194–(–12); *P* < 0·001) as well as for White non-Hispanic (–99 mcg; IQR –167–(–41); *P* < 0·003) and Black non-Hispanic (–189 mcg; IQR –222–(–9) ; *P* < 0·021) participants (Table 3). Of note, average natural folate without supplementation was measured at half the amount at baseline (316 mcg) and endpoint (252 mcg).

**Table 1:** Participants baseline characteristics (*n* 32)

Variable	*n*	%	Mean	sd
Age (years)			26·8	4·3
Female	32	100 %		
Race/ethnicity				
White non-Hispanic	11	34 %		
Black non-Hispanic	11	34 %		
Hispanic	10	32 %		
Health insurance				
Private	31	97 %		
Public	1	3 %		
ADI state index (*n* 30)	3 ± 1·36 (1–6)			
ADI national index (*n* 30)	53 ± 17 (12–81)			
Highest level of education				
Actively enrolled in a 4-year college	2	6·3 %		
Bachelor’s degree	10	31·3 %		
Master’s degree	17	53·1 %		
Professional degree	3	9·4 %		
Annual income level				
$0–< $20 000	15	46·9 %		
$20 000–< $40 000	10	31·3 %		
$40 000–< $60 000	4	12·5 %		
$60 000–< $80 000	1	3·1 %		
$80 000–< $100 000	2	6·3 %		
Currently employed				
No	12	37·5 %		
Yes – part-time	9	28·1 %		
Yes – full time	11	34·4 %		
Presence of Crohn’s disease	0			
Presence of sickle cell anaemia	1	3 %		
Diagnosis of NTD	0			
Family history of NTD	0			
Erythrocyte folate (nmol/l)			1806	337
Vitamin B_12_ (pmol/l)			410	161

ADI, area deprivation index: 1-least disadvantaged; 10-most disadvantaged; NTD, neural tube defect.

**Table 2:** Diet characteristics of the study participants (*n* 32)

Variables	Number of participants (%)			
Vitamins (daily)	Baseline		Endpoint	
	*n*	%	*n*	%
Multivitamin	13	41 %	10	32 %
Vitamin B_12_	1	3 %	1	3 %
Biotin	2	6 %	3	9 %
Types of specific diets a participant follows				
Low carbohydrate (Atkins, Paleo, etc.)	2	6 %	2	6 %
Ketogenic	0		0	
Vegan	0		0	
Vegetarian	1	3 %	1	3 %
Gluten-free	2	6 %	2	6 %
Low fat	2	6 %	2	6 %
Low Na	1	3 %	0	
Low calorie	2	6 %	0	
Weight watchers	0		0	
Diabetic	0		1	3 %
Intermittent fasting	3	9 %	2	6 %
Dietary approaches to stop hypertension	0		0	
Mediterranean	2	6 %	2	6 %
Other	3	9 %	1	3 %
None	21	66 %	24	75 %
	Mean	sd	Mean	sd
Total daily energy intake	2018	690	1575	654

**Table 3:** Total dietary folate intake by race and ethnicity (*n* 32)

Race/ethnicity	Baseline dietary folate intake (mcg)				Endpoint dietary folate intake (mcg)				Difference (mcg)				95 % CI	*P*-value^*^
	Mean	sd	Median	IQR	Mean	sd	Median	IQR	Mean	sd	Median	IQR		
Total (*n* 32)	657	330	574	372–887	545	319	438	299–866	−111	195	−94	–191–(–12)	−182, 41	0·001
White non-Hispanic (*n* 11)	660	356	509	357–887	561	318	443	307–847	−99	73	−61	–167–(–41)	−0·15, 49	0·003
Black non-Hispanic (*n* 11)	703	382	643	454–1065	517	355	428	290–878	−186	281	−189	–222–(–9)	−375, 2·66	0·021
Hispanic (*n* 10)	602	257	571	353–857	558	309	494	237–877	−44	161	−63	–186–89	−158, 71	0·386

Red colour shows significance (< 0.05).

* Wilcoxon signed rank two-tailed paired sample test.

**Fig. 2:**
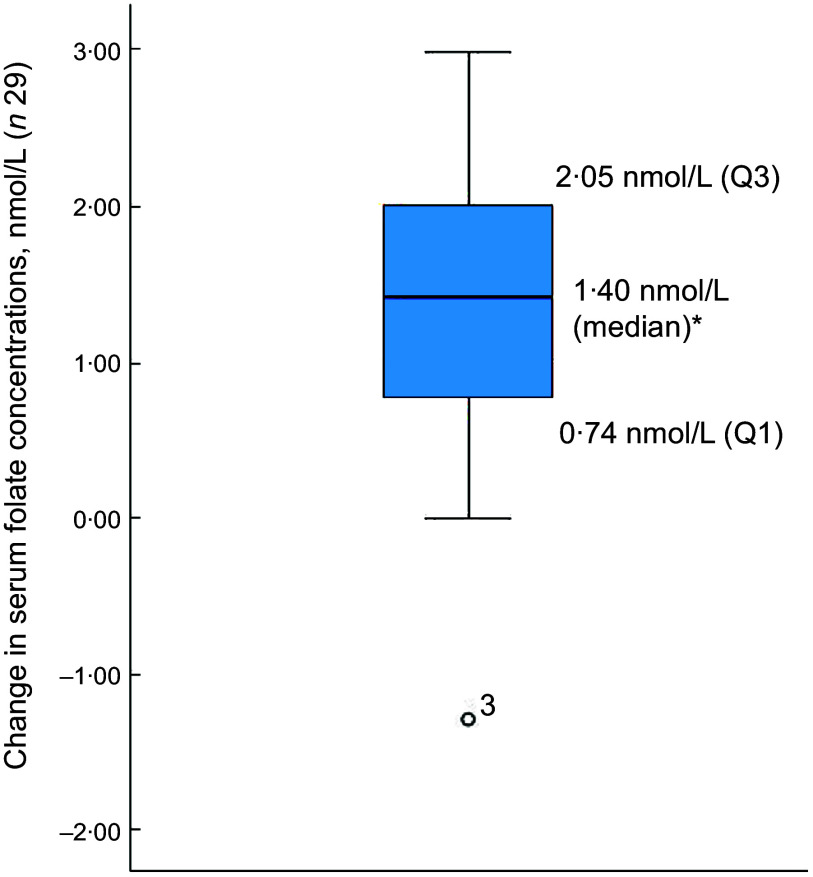
Boxplot of change in serum folate concentrations between baseline and endpoint *Wilcoxon signed rank test (*P* < 0·001); ^o^ is the outlier

**Fig. 3:**
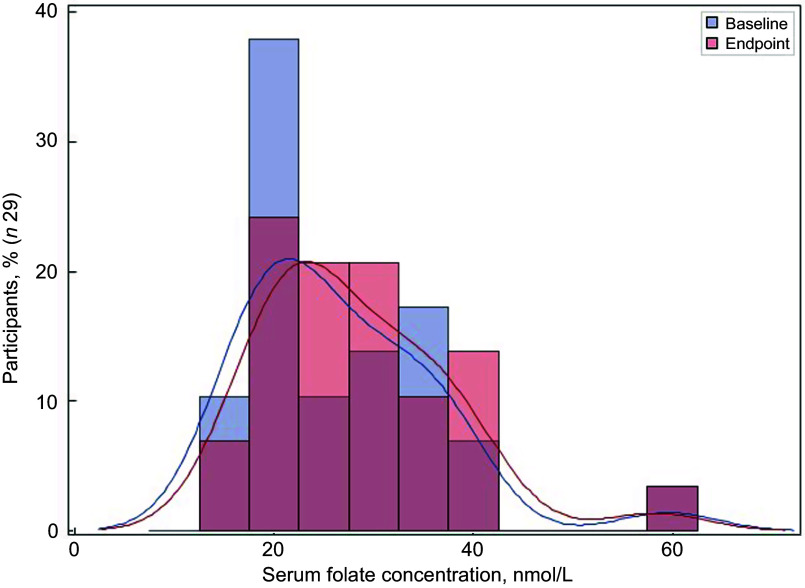
Distribution of the serum folate concentrations at baseline and endpoint

**Fig. 4:**
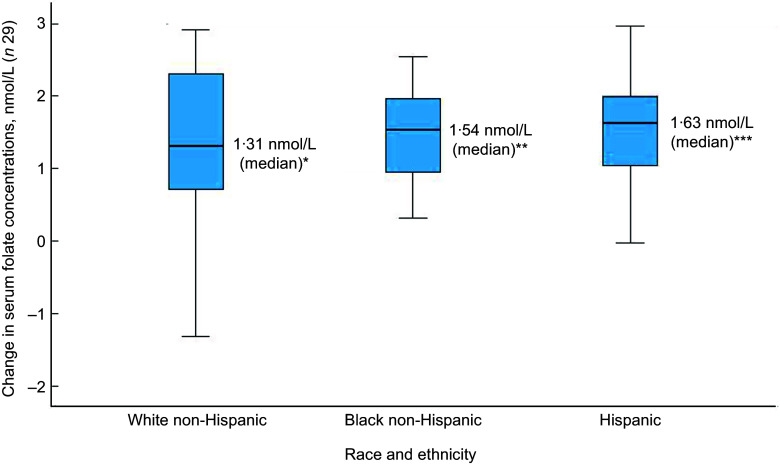
Boxplot of change in serum folate concentrations by race and ethnicity Wilcoxon signed rank two-tail paired sample test (*P < 0·033; **P < 0·005; ***P < 0·007)

Of the thirty-two participants, three had folate levels above 90th of which some had in the pre (*n* 2) or the post (*n* 3) intervention period and were excluded from the analysis for the primary outcome. This narrowed our analysis to twenty-nine paired samples on which this analysis is based. Overall, twenty-eight of twenty-nine women (93 %; 95 % CI 0·77, 0·99) saw an increase in their folate concentration post-intervention. For this analysis sample of twenty-nine participants, the Shapiro–Wilk test for normality showed skewed distribution at the baseline (*P* < 0·003) and endpoint levels (*P* < 0·013). Results of our analysis for the primary outcome of the study showed a mean folate increase of 1·43 (sd 0·95) nmol/l (95 % CI 1·07, 1·79; *P* < 0·001) from 26·78 (sd 9·66) nmol/l (baseline) to 28·21 (sd 9·30) nmol/l at the study endpoint (Table 4). The median folate concentrations increased from 24·08 nmol/l (baseline) to 25·96 nmol/l (endpoint) with a median increase of 1·40 nmol/l (IQR 0·74–2·05; *P* < 0·001; Fig. 2). Figure 3 shows the statistically significant right shift from pre-intervention to post-intervention, indicating improved serum folate concentrations. The statistically significant increase was seen across all three race/ethnic groups: White non-Hispanic 1·31 nmol/l (IQR 0·58–2·39; *P* < 0·033), Black non-Hispanic 1·54 nmol/l (IQR 0·84–2·01; *P* < 0·005) and Hispanic 1·63 nmol/l (IQR 0·92–2·04; *P* < 0·007) (Table 4, Fig. 4). Spearman correlation test did not show a significant correlation between the amount of consumed folic acid through FISFA and the change in serum folate concentrations (rho = 0·21; 95 % CI –0·18, 0·54; *P* = 0·272).

**Table 4:** Serum folate concentrations pre- and post-FISFA intervention among women of reproductive age (18–40 years) (*n* 29)

Race/ethnicity	Baseline serum folate levels (nmol/l)				Endpoint serum folate levels (nmol/l)				Difference (nmol)				95 % CI	*P*-value^†^
	Mean	sd	Median	IQR	Mean	sd	Median	IQR	Mean	sd	Median	IQR		*P*-value^*^
Total (*n* 29)	26·78	9·66	24·08	19·67–33·68	28·21	9·30	25·96	21·60–35·07	1·43	0·95	1·40	0·74–2·05	1·07, 1·79	0·001
														0·001
White non-Hispanic (*n* 9)	30·49	13·43	28·95	20·86–37·01	31·77	12·91	31·87	22·33–38·53	1·28	1·31	1·31	0·58–2·39	0·28, 2·29	0·033
														0·019
Black non-Hispanic (*n* 10)	23·73	7·51	19·74	17·78–30·01	25·21	7·02	21·71	19·75–31·66	1·48	0·72	1·54	0·84–2·01	0·96, 1·99	0·005
														0·001
Hispanic (*n* 10)	26·48	7·02	25·79	21·67–32·00	28·00	6·99	26·78	23·47–32·74	1·51	0·86	1·63	0·92–2·04	0·90, 2·13	0·007
														0·001

FISFA, fortified iodised salt with folic acid.

The conversion rate of folate from ng/ml to nmol/l is 2·265 per WHO 2015 guidance^(37)^.

Red colour shows significance (< 0.05).

* Wilcoxon signed rank two-tailed paired sample test.

† Two-tailed paired sample *t* test.

Our secondary objective, examining study feasibility, showed that all eligible subjects (*n* 32) consented and enrolled in the study met the study endpoint with complete data. Regarding salt acceptability as a secondary objective, on average, participants consumed FISFA for 24·81 (sd 6·6) d (median 25·5 d), with a mean daily intake of 1·27 g (sd 0·92) g (range 0·2–3·42 g, median 1·08 g). We observed a wide range of total FISFA consumption, at 32·65 g (sd 27·3) g (range 4·96–102·52 g, median 27·17 g) (Table 5). FISFA consumption was measured both by completion of daily salt logs and by weighing saltshakers before and after the study. As FISFA salt has a light yellow tinge, the acceptability was measured in terms of the perception of taste and colour of the salt. Acceptability of FISFA was high regarding both taste and colour (96·7 % and 90 %, respectively). Over 81·3 % responded ‘strongly disagree’ and 15·6 % ‘disagree’ to ‘Did using this salt negatively affect the taste of your food overall?’ Similar responses were seen regarding the colour of the salt at 68·8 % ‘strongly disagree’ and 21·9 % ‘disagree’ when asked, ‘Did the colour of salt negatively affect your desire to use it daily?’

**Table 5:** Feasibility and acceptability of FISFA among study participants (*n* 32)

Variable	*n*	%	Mean	sd	Median	Range
Feasibility						
Consent rate	32	100 %				
Follow-up completion	32	100 %				
Survey completion	32	100 %				
Acceptability						
Daily amount of FISFA consumption (g)	1·27g ± 0·92		1·27	0·92	1·08	0·2–3·42
Total amount of FISFA consumption (g)	32·65g ± 27·31		32·65	27·31	27·7	4·96–102·52
Number of days of FISFA consumption (d)	24·81 ± 6·6		24·81	6·6	25·5	7–35
FISFA negatively affected the taste of food overall						
Strongly disagree	26	81·3 %				
Disagree	5	15·6 %				
Neither agree nor disagree	1	3·1 %				
Agree	–					
Strongly agree	–					
The colour of FISFA negatively affected the desire to use it daily						
Strongly disagree	22	68·8 %				
Disagree	7	21·9 %				
Neither agree nor disagree	2	6·3 %				
Agree	1	3·1 %				
Strongly agree	–					

FISFA, fortified iodised salt with folic acid.

## Discussion

To our knowledge, this pilot project reports the first clinical data assessing whether salt fortified with folic acid further improves serum folate concentrations for a country with a mandatory and effective folic acid-enriched grain product fortification policy.

Our data show a statistically significant increase in serum folate levels after simply substituting a saltshaker with folic acid for 1 month. We see an increase in nearly all study participants after consuming FISFA. The median increase is similar when comparing groups by race/ethnicity, supporting the notion that FISFA could be a universal vehicle and could influence races/ethnicities equally. We infer that there is still room for improvement even in the USA regarding current folate intake. Continued intake of FISFA for up to 6 months would allow assessment of erythrocyte as an indicator of long-term intake. We believe the dosage of 100 mcg per 1 g of salt was adequate in the setting of a saltshaker. However, in the overall goal of adding folic acid to all salt used in prepackaged processed foods, cooking salt and table salt, the dosing should account for the current Na intake in the USA, which is estimated to be between 3·3 g and 9·2 g a day and adjusted accordingly. Although we did not find a strong correlation between FISFA intake and serum change, this could be explained by a small number of participants and a short study period. Additionally, the assessment of insufficiency status in the Southeastern US should be checked using the established microbiological assay method.

NHANES cross-sectional data from several studies between 1988 and 2016 show an almost threefold increase in serum folate levels from 10·8 nmol/l before flour fortification to 36 nmol/l after fortification with a mean difference of 24 nmol/l. Specifically, the USA saw an increase from 10·8 nmol/l in the period of 1988–1994 to 29·5 nmol/l in 1999–2000, with a further increase from 34 nmol/l in 2007–2010 to 36 nmol/l in 2011–2016 in WRA^(18,33)^. The median and mean serum folate levels at baseline found in our study are comparable with the reported mean serum level of 36 nmol/l in the earlier NHANES study using a nationally representative sample, although we acknowledge the use of different assay methods^(18)^.

Further, our study shows encouraging results regarding FISFA acceptability regarding taste and colour. In addition, all enrolled participants followed the study protocol and completed follow-up.

In regard to dietary folate intake, our data show similar amounts of dietary intake of folate from diet and from supplements at baseline, as reported in other studies^(19,38)^. We observe a decrease in folate intake overall at the endpoint compared with the baseline visit, which could be due to recall bias or overestimation of food consumption at baseline that aligns with the observed decrease in total calorie intake from baseline to the follow-up visit. However, it was interesting to note an overall increase in the serum folate concentrations in the setting of dietary folate decrease, which we cannot readily explain using our study variables. Further research could be done to test the hypothesis that FISFA not only increased serum levels but also compensated for the potential decrease in dietary folate. We also see a potential trend with Hispanic and White non-Hispanic women consuming less dietary folate at baseline. This may suggest the need for additional food vehicles like salt to adapt to various cultural food preferences.

The USA has made substantial progress in reducing NTD cases, but worldwide, modelled data from the year 2020 show that only about 22 % of folate-preventable spina bifida is being prevented through mandatory fortification of wheat flour, maize flour and/or rice in about sixty countries that implement the intervention^(31)^. There are challenges to grain fortification in regions that grow food locally or that lack centralised processing of grains to facilitate large-scale fortification^(39)^. Therefore, we suggest that salt has the potential to be a very good vehicle for global fortification efforts since it is in every household, it is not grown by individual families, and most importantly, there is already global iodisation of salt, which provides the infrastructure to add one other micronutrient to the process with little cost. From the global perspective, a recent modelling study based on data from 100 countries indicated that salt fortification with folic acid could achieve the prevention of an additional 65 % of NTD^(40)^.

We believe more research is needed on the effectiveness and dosage of folic acid in salt to help account for WHO recommendations to lower Na intake^(41)^. One concern that has arisen in reviewing these protocols is that FISFA could increase salt intake in the population, which could lead to hypertension problems. This study does not advocate for an increase in salt intake among pregnant women and in the overall population but, rather, is motivated by an interest in substituting currently ingested salt products from processed foods, cooking and table salt with FISFA. Concentrations of folic acid in FISFA can be adjusted to meet public health recommendations on lowering Na intake in the general population. Further, the proposed approach of fortified salt with folic acid could complement the existing flour fortification. It could also potentially help address the lack of reach of wheat flour fortification observed in some US-Hispanic communities which puts them at a higher risk of NTD-affected pregnancy.

Our study was conducted in the USA, which has had mandatory fortification of flour with folic acid since 1998. Elsewhere, a recent study conducted in a rural region of India showed a significant median increase of 39·8 nmol/l in folate concentrations among non-pregnant non-lactating WRA (*n* 83)^(26)^. Although this used a similar pre- and post-intervention study design to look at folate levels in women who consumed double-fortified salt (folic acid + iodine), India has no existing FAF policy in the country^(26)^. Our study is thus valuable and novel and suggests that a simple modification in the additional source of folic acid could potentially increase serum folate concentrations further. This is especially important since most recent NHANES data showed additional FAF is key to have a sufficient protective level of folate for women who only consume folic acid from enriched cereal grain products^(19)^. This is also recognised by the US Preventive Services Task Force that recommends additional folic acid intake in WRA^(35)^. Furthermore, the study demonstrates the feasibility that salt fortification could complement flour fortification.

Although assessing serum folate insufficiency was not a goal of our study, readers may wonder about the implications associated with an increase in folate concentrations and how it is connected to NTD risk. As mentioned earlier, there is an established serum folate cut-off point of 25·5 nmol/l as a protective sufficiency level against developing a fetus with an NTD. This cut-off point was established using a microbiological assay method. Our study used an immunoassay that does not yet have an established insufficiency cut-off point. Due to the lack of standardisation of folate assays globally and the use of different types of assays for folate analysis, this has led to issues with different cut-off points for insufficiency levels^(42–44)^. Lack of traceability and communicability to standard reference materials and methods is a common and recurring issue in clinical science, particularly in longitudinal population studies where laboratory assays and standardisation change over time. If we cautiously apply mismatched microbiological assay cut-off points to our immunoassay results, we found serum insufficiency in 15/32 (47 %) participants, which is more than double the nationally reported percentage (20 %)^(18)^. This difference may be due to participant characteristics in our small number of study participants in comparison to the nationally representative NHANES data, as well as the application of mismatched cut-off points using a different analysis method. Therefore, we are focusing our results on whether FAF in salt is successful in raising circulating folate concentrations or not (i.e. proof of concept), rather than focusing on specific cut-off points of insufficiency.

Our study had several strengths and limitations. Key strengths included high adherence to the study protocol by the participants, complete follow-up and complete data collection. Participants were monitored weekly, adhered to not sharing the salt with others and did not have live-in children, all of which helped minimise risks of external influences that could affect study outcomes. Another strength was the ability to measure the weight of the salt before and after as a measure of salt consumption. Study limitations included the recruitment of all participants at a single site (one geographic location), which may challenge external validity. Also, the average consumption of Na in Western diets ranges from 8 to 10 g, and most Na in the USA is consumed from processed and prepackaged foods^(45)^. Even allowing for this limitation, however, there is a potential for improving folate levels with small changes introduced via a simple saltshaker substitution.

In conclusion, this novel pilot study showed a small yet meaningful increase in serum folate concentrations in WRA after 1 month of voluntary consumption of FISFA in a setting of mandatory fortification with folic acid of enriched grain products. The study was deemed feasible to conduct in the Southeastern US FISFA taste and colour proved highly acceptable to study participants. FISFA could potentially complement current fortification efforts in the USA to increase folate levels if implemented in all salt used in processed foods, cooking salt and table salt. Future studies should replicate findings in a larger sample. The recent World Health Assembly resolution on food fortification urges countries to implement FAF to prevent NTD. Double-fortified salt with iodine and folic acid may hold promise to address gaps in the global prevention of spina bifida and anencephaly, especially in many low- and middle-income countries with existing successful salt iodisation programmes.
